# Systematic evaluation, verification and comparison of tuberculosis‐related non‐coding RNA diagnostic panels

**DOI:** 10.1111/jcmm.15903

**Published:** 2020-12-13

**Authors:** Mengyuan Lyu, Yuhui Cheng, Jian Zhou, Weelic Chong, Yili Wang, Wei Xu, Binwu Ying

**Affiliations:** ^1^ Department of Laboratory Medicine West China Hospital Sichuan University Chengdu China; ^2^ West China School of Medicine Sichuan University Chengdu China; ^3^ Department of Thoracic Surgery West China Hospital Sichuan University Chengdu China; ^4^ Sidney Kimmel School of Medicine Thomas Jefferson University Philadelphia PA USA; ^5^ Department of Biostatistics Princess Margaret Cancer Centre University Health Network Toronto ON Canada; ^6^ Dalla Lana School of Public Health University of Toronto Toronto ON Canada

**Keywords:** diagnostic panel, ncRNAs, performance, tuberculosis

## Abstract

We systematically summarized tuberculosis (TB)‐related non‐coding RNA (ncRNA) diagnostic panels, validated and compared panel performance. We searched TB‐related ncRNA panels in PubMed, OVID and Web of Science up to 28 February 2020, and available datasets in GEO, SRA and EBI ArrayExpress up to 1 March 2020. We rebuilt models and synthesized the results of each model in validation sets by bivariate mixed models. Specificity at 90% sensitivity, area under curve (AUC) and inconsistence index (*I* ^2^) were calculated. NcRNA biofunctions were analysed. Nineteen models based on 18 ncRNA panels (miRNA, lncRNA, circRNA and snoRNA panels) and 18 datasets were included. Limited available datasets only allowed to evaluate miRNA panels further. Cui 2017 and Latorre 2015 exhibited specificity >70% at 90% sensitivity and AUC >80% in all validation sets. Cui 2017 showed higher specificity at 90% sensitivity (92%) and AUC (95%) and lower heterogeneity (*I* ^2^ = 0%) in ethological‐confirmation validation sets. Gene Ontology and Kyoto Encyclopedia of Genes and Genomes analysis indicated that most ncRNAs in panels involved in immune cell activation, oxidative stress, and Wnt and MAPK signalling pathway. Cui 2017 outperformed other models in both all available and aetiological‐confirmed validation sets, meeting the criteria of target product profile of WHO. This work provided a basis for clinical choice of TB‐related ncRNA diagnostic panels to a certain extent.

## 1 INTRODUCTION

Accurate diagnosis and effective treatment hold the key to interrupting tuberculosis (TB) transmission. The whole world is successfully narrowing the gap between TB incidence and treatment proportion. The Global TB Report 2019 indicates that global TB treatment coverage increases to 69% in 2018, and treatment success increases to 85% in 2017.^1^ Most TB patients could receive effective therapy as long as they get diagnosed timely. Early and precise diagnosis is essential to mitigate TB burden. Currently, two ways are used for TB detection: one aims to detect bacteria itself, mycobacterium tuberculosis (MTB); another targets at specific biomarkers of host immune response. However, up to 50% TB patients are considered as bacteriological‐negative TB; that is, clinical symptoms, imaging features and response to anti‐TB treatment support the diagnosis of TB but there is no aetiological evidence, even when relatively sensitive nucleic acid testing is used.^2^ Long culture time, high equipment requirements and unqualified sputum sample quality limit the diagnostic performance and widely use of pathogen‐based detection.^3^ World Health Organization (WHO) recommends a non‐sputum‐based detection should have at least 90% of sensitivity and 70% of specificity when compared with confirmatory tests.^4^ Nevertheless, current non‐sputum‐based detections, which measure biomarkers of host immune responses, have insufficient diagnostic capacity.^5^ Novel diagnostic methods are urgently needed.

Non‐coding RNAs (ncRNAs) mainly include microRNAs (miRNAs), long non‐coding RNAs (lncRNAs), circular RNA (circRNAs), PIWI‐interacting RNAs (piRNAs), small nucleolar RNAs (snoRNAs) and small nuclear RNA (snRNA), etc^6^ NcRNAs occupy nearly 60% of the transcriptional output in human cells.^6^ Advances in technologies and researches reverse our misperception that ncRNAs with low transcriptional potential are useless molecules and confirm ncRNAs participate in many pathophysiological processes including various cellular functions and post‐transcriptional regulation in eukaryotes.^7^, ^8^ Abundant biological functions, ability to reflect disease progression and tissue‐ or time‐specific expression contribute ncRNAs to being considered as the next paradigm shift in disease diagnosis.^9^, ^10^, ^11^

The roles of ncRNAs played in TB also have been reported,^12^, ^13^, ^14^ and then, several ncRNAs diagnostic panels for TB have appeared, with improved diagnostic and predictive performance.^3^, ^15^ These panels serve different clinical purposes, including distinguishing active TB patients from healthy controls (HCs) or latent TB infection (LTBI), and predicting TB progression. However, these panels are not widely applied to the clinic. Of noting, different panels use different sample types (whole blood (WB), serum, etc), ncRNA types (miRNAs, lncRNAs, etc) and modelling methods (logistic regression, linear combination, etc). Clinicians are confused to choose an optimal panel due to the diversity of these panels. Besides, most ncRNA panels are selected and validated by participants of the same ethnicity, and thus, the robustness and generalizability of these panels are unclear. It is hard to guarantee the capacity of these panels in different situations. Moreover, relevant studies have not provided proper approaches to tailor the structure of ncRNA panels to meet corresponding needs. Furthermore, detecting multiple ncRNAs simultaneously in a panel is still technically challenging.^16^ Both the number of ncRNAs in panels and diagnostic performance should be carefully considered.

TB‐related host response gene diagnostic signatures (ie based on coding RNAs) have been systematically evaluated.^17^ However, to our knowledge, no systematic assessment of ncRNA diagnostic panels in TB has been reported. Here, we (i) systematically evaluated published ncRNA diagnostic panels, relevant available microarray and sequencing datasets; (ii) implemented a classification model and validated the performance of each model in eligible datasets; and (iii) explored the scope of applying each panel by subgroup analyses and function analyses. We aimed to identify an optimal ncRNA panel with excellent performance and generalizability and thus offer some support for clinical choice.

## 2 MATERIALS AND METHODS

**FIGURE 1:**
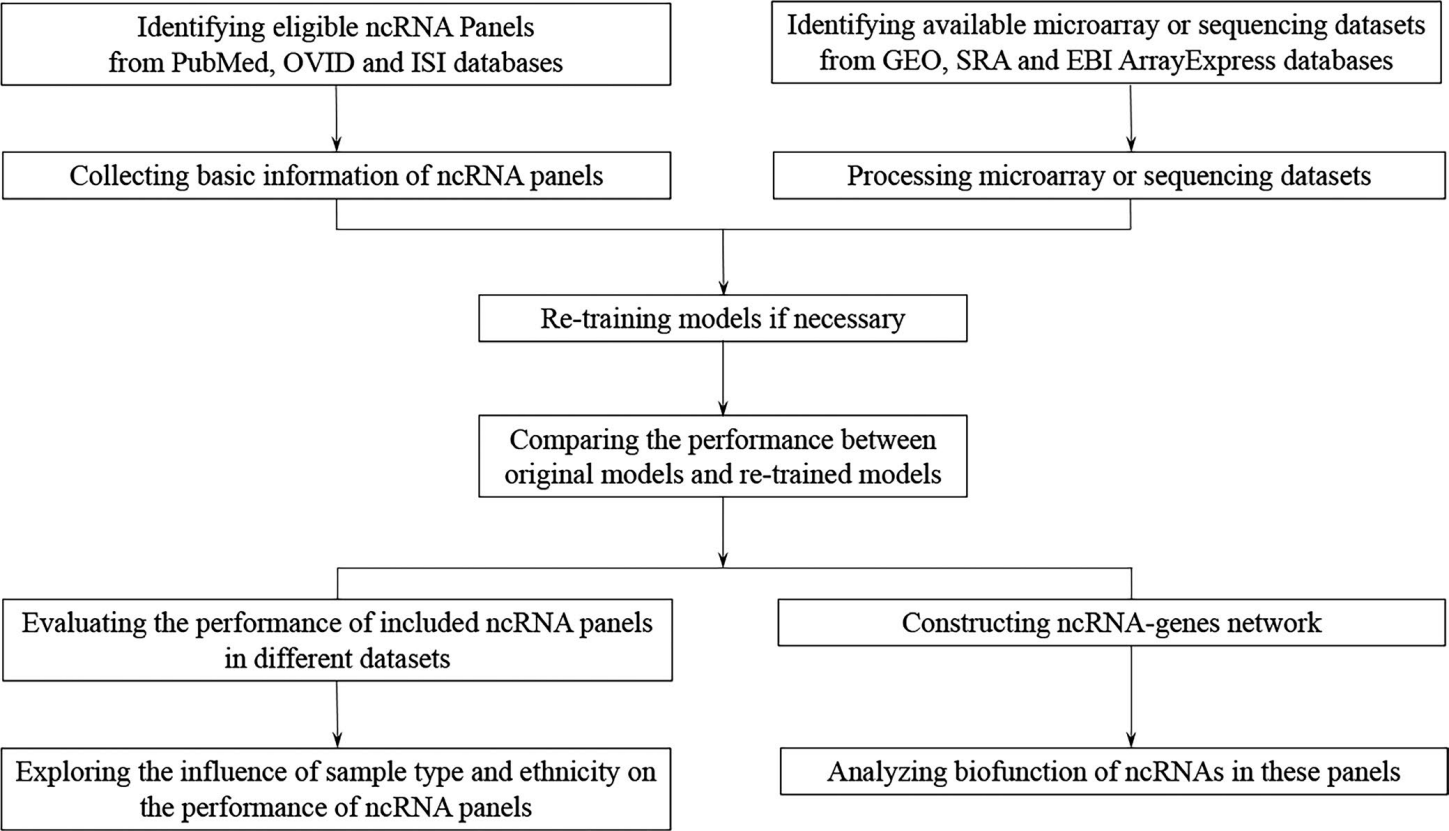
The flow chart of study design. ncRNA, non‐coding RNA; SRA, Sequence Read Archive; EBI, European Bioinformatics Institute

The design of this work is shown in Figure 1.

### 2.1 Collecting ncRNA panels

To identify eligible ncRNA panels, we searched PubMed, OVID and Web of Science from database inception up to 28 February 2020. We limited the species to *Homo sapiens*, but not study type or language. The search terms included TB (tuberculosis) AND diagnosis (“diagnose” OR “diagnostic” OR “panel” OR “signature” OR “combination” OR “profile”) AND ncRNA (“non‐coding RNA” OR “miRNA” OR “microRNA” OR “lncRNA” OR “long non‐coding RNA” OR “circular RNA” OR “circRNA” OR “PIWI‐interacting RNA” OR “piRNA” OR “small nucleolar RNA” OR “snoRNA” OR “small nuclear RNA” OR “snRNA”). Reference lists of relevant studies and articles which cited relevant studies as references were also reviewed. We only included articles which constructed ncRNA panels to diagnose TB based on peripheral blood or its components, but not studies focusing on the diagnostic performance of signal ncRNA (see Figure S1).

Two investigators (Lyu M and Cheng Y) independently undertook the work of search, data extraction and assessment of modelling quality based on Transparent Reporting of a multivariable prediction model for Individual Prognosis Or Diagnosis (TRIPOD),^18^ and disagreements would be discussed with a third investigator (Zhou J).

### 2.2 Identifying eligible microarray and sequencing data

We searched public databases including NCBI GEO, NCBI Sequence Read Archive (SRA) and European Bioinformatics Institute (EBI) ArrayExpress on 1 March 2020, with the terms of TB or its full name AND non‐coding RNA or its alternative terms, as described above. We did not restrict detection methods and platforms. We included studies using peripheral blood or its components, but excluded studies using cultured human blood cells infected with MTB in vitro such as GSE94007 and GSE145770. The expression profiles of ncRNAs in cells grown in vitro were different from those in vivo due to the complex and delicate regulatory mechanisms in human bodies.^19^

### 2.3 Processing microarray and sequencing data

For raw data in SRA, Trimmomatic 0.39 was used to obtain clean reads by deleting raw reads below 20 bp in 5ʹ and 3ʹ ends, filtering bases below quality 20 and removing reads of length below 17 after processing. Quality control was conducted by FastQC (Babraham Bioinformatics), and the filtered reads would be processed. For example, miRDeep 2.0.1.2 was applied to identify and quantify of the filtered reads based on the sequence of 1917 precursor miRNAs and 2656 mature miRNAs.

We applied k‐nearest neighbours algorithm to impute missing values in expression matrix^20^ and further assess imputation accuracy.

When necessary, data in each dataset were normalized by limma voom and log_2_ base transformed. Sequencing and microarray data were both included. Package sva of R (R Foundation for Statistical Computing; version 3.6.2.) was used to adjust butch effect, and the following formula was applied for normalization.

$$F_{\mathrm{ij}}=\frac{E_{\mathrm{ij}}}{\frac{\sum_{j=1}^{n}E_{\mathrm{ij}}}{n}}$$

where *n* was the number of samples in the datasets. *F_ij_* represented the expression of ncRNA_i_ on sample *j* (*E_ij_*) divided by the average of the expression of ncRNA*_i_* in all the samples.

### 2.4 Model rebuilding

Included models would be rebuilt by R if necessary. In order to reproduce model exactly, the same modelling method and parameters in original articles were used (Text S1). If available, we trained the model with the original data or similar datasets. We compared diagnostic performances between the rebuilt models and original models to ensure the accuracy of rebuilding (Table S1). The remaining available datasets were treated as validation sets. We excluded polymerase chain reaction (PCR) data to maintain consistency between training set and validation sets and keep sufficient dataset coverage.

### 2.5 Validation of model performance

To comprehensively assess the applicability of each model, each included model was validated in all eligible datasets. If the dataset did not include any variables in models, this dataset would be excluded. Sensitivity, specificity and area under curve (AUC) of each model in each dataset were generated. To avoid bias, we removed training set of each model from the corresponding validation cohorts. The cut‐off value to determine positive diagnostic test results was calculated by the Youden index of each model in their own training set.

### 2.6 Bivariate meta‐analysis

Bivariate mixed models were taken to pool the results^21^ by midas in Stata 15.1. We pooled sensitivity, specificity, positive predictive value (PPV), negative predictive value (NPV) and AUC, each with a 95% confidence interval (CI). According to the criteria of the target product profile (TPP) provided by WHO,^4^ specificity at 90% sensitivity was also examined. The heterogeneity across different datasets was quantitatively evaluated by Higgin's *I* ^2^, with *I* ^2^ > 50% denoting significant heterogeneity.^22^ Meta‐regression was conducted to explore the sources of heterogeneity and application scope for each model.

### 2.7 Biofunction of ncRNAs in panels

MicroRNA Data Integration Portal (mirDIP) 4.1.11.1 and the Encyclopedia of RNA Interactomes (ENCORI) were used to predict targeted genes of miRNAs and lncRNAs, respectively. The parental genes of circRNAs were provided by circBase. The Gene Ontology (GO) and Kyoto Encyclopedia of Genes and Genomes (KEGG) were applied to show functions of targeted or parental genes.

## 3 RESULTS

### 3.1 Basic information of ncRNA panels and available datasets

We included 18 articles, with 18 ncRNA panels and 19 ncRNA diagnostic models (Table 1). We analysed 13 miRNA diagnostic models (Miotto 2013 was developed by two different methods to build models based on one panel),^3^, ^32^ one model that combined miRNA and snoRNA,^33^ one lncRNA model^34^ and four circRNA models.^35^, ^36^, ^37^, ^38^ We also performed quality assessment of included articles (Table 2).

**TABLE 1:** The characteristics of included panels

Author Year	Ref^a^	Participants region	Mean age (years)	HIV^+^ number	Treatment status	TB^b^ diagnostic method	Sample type	Original dataset	Training set	Model purpose	NcRNA^c^ type	NcRNA number	Modelling method	Model rebuilding
Latorre 2015	23	Southern Europe	NA^d^	NA	NA	Culture	WB^e^	NA	GSE29190	TB vs (HC^f^ and LTBI^g^)	MiRNA^h^	4	Linear kernel SVM^i^	Yes
Pan 2019	15	East Asia	≥18	0	NA	Culture, smear or Xpert	PBMC^j^	GSE131708	GSE131708	TB vs (HC and DC^k^)	MiRNA	4	Logistic regression with forward stepwise	Yes
Wang 2011	24	East Asia	≥18	0	None	Smear or radiology	PBMC	GSE29190	GSE29190	TB vs (HC and LTBI)	MiRNA	17	SMV	Yes
Zhou 2016	25	East Asia	<18	0	NA	Comprehensive diagnosis	PBMC	NA	GSE34608 ^†^	TB vs HC	MiRNA	8	Logistic regression	Yes
Barry 2018	26	East Asia	>18	0	None	Comprehensive diagnosis	Plasma	NA	GSE116542	TB vs HC	MiRNA	5	Logistic regression	Yes
Cui 2017	27	East Asia	NA	0	NA	NA	Plasma	NA	GSE116542	TB vs HC	MiRNA	3	Linear combination	No
Duffy 2018	28	South and East Africa	>18	0	Some	Culture or smear	Serum	NA	GSE116542	TB vs household contacts	MiRNA	47	Elastic‐net logistic regression	Yes
Miotto 2013‐RVM/AIC logistic regression	29	Southern Europe and east and south Africa	≥18	4	NA	Culture, smear or Xpert	Serum	NA	GSE116542	TB vs HC	MiRNA	15	RVM^l^ AIC^m^ logistic regression	Yes Yes
Qi 2012	30	East Asia	>18	0	None	Culture and smear	Serum	NA	GSE116542	TB vs HC	MiRNA	3	Logistic regression	Yes
Zhang 2013	31	East Asia	>18	0	None	Symptom, culture and radiology	Serum	SRP029907	GSE116542 ^‡^	TB vs HC	MiRNA	6	Logistic regression	Yes
Alipoor 2019	32	West Asia	≥15	0	NA	Culture, smear and PCR^n^	Exosome	NA	GSE116542	TB vs HC	MiRNA	3	Logistic regression	Yes
Hu 2019	3	East Asia	>18	0	None	Culture	Exosome	GSE116542	GSE116542	TB vs HC	MiRNA	6	Linear kernel SVM	Yes
de Araujo 2019	33	South America	>18	NA	Some	Comprehensive diagnosis	WB	GSE131174	GSE131174	TB vs (HC and LTBI)	MiRNA and snoRNA^o^	4	SVM	Yes
Chen 2017	34	East Asia	≥18	0	None	Comprehensive diagnosis	Plasma	NA	GSE101805 ^§^	TB vs HC	LncRNA^p^	4	Logistic regression	Yes
Huang 2018	35	East Asia	>18	0	NA	Culture, smear or other aetiological evidence	PBMC	NA	GSE117563	TB vs HC	CircRNA^q^	2	Logistic regression	Yes
Qian 2018	36	East Asia	>18	0	NA	Comprehensive diagnosis	PBMC	GSE103188	GSE103188	TB vs HC	CircRNA	7	Linear combination	No
Huang 2018	37	East Asia	>18	0	NA	Culture or smear	Plasma	NA	GSE106953	TB vs HC	CircRNA	2	Logistic regression	Yes
Huang 2018	38	East Asia	>18	0	NA	Culture, smear or other aetiological evidence	Plasma	NA	GSE106953	TB vs HC	CircRNA	2	Logistic regression	Yes

Note

a: reference; b: tuberculosis; c: non‐coding RNA; d: non‐available; e: whole blood; f: healthy control; g: latent tuberculosis infection; h: micro RNA; i: support vector machine: j: peripheral blood mononuclear cell; k: disease control; l: relevance vector machine; m: Akaike information criterion; n: polymerase chain reaction; o: small nucleolar RNA; p: long non‐coding RNA; q: circular RNA.

^†^ The data of tuberculosis patients and healthy controls selected from GSE34608 were used as training set.

^‡^ Zhang et al used sequencing data of 20 samples as training set and provided the information of their training set (SRP029907); however, we only found the data of 2 samples in SRA database which was not enough to support model reconstruction. Thus, GSE116542 was used as training set.

^§^ The data of tuberculosis patients and healthy controls in GSE101805 were used as training set.

**TABLE 2:** The assessment of included articles according to TRIPOD^a^

Item	Development or validation?	Checklist item	Latorre 2015	Pan 2019	Wang 2011	Zhou 2016	Barry 2018	Cui 2017	Duffy 2018	Miotto 2013	Qi 2012	Zhang 2013	Alipoor 2019	Hu 2019	de Araujo 2019	Chen 2017	Huang 2018	Qian 2018	Huang 2018	Huang 2018
1	Development	Identify the study as developing and/or validating a multivariable prediction model, the target population, and the outcome to be predicted	Yes	Yes	No	Yes	No	Yes	Yes	Yes	Yes	Yes	No	Yes	Yes	Yes	No	Yes	Yes	Yes
2	Development	Provide a summary of objectives, study design, setting, participants, sample size, predictors, outcome, statistical analysis, results, and conclusions	No	Yes	No	No	Yes	Yes	No	Yes	No	Yes	No	Yes	Yes	No	Yes	No	No	Yes
3a	Development	Explain the medical context (including whether diagnostic or prognostic) and rationale for developing or validating the multivariable prediction model, including references to existing models	Yes	Yes	No	No	Yes	Yes	Yes	No	Yes	No	Yes	Yes	No	No	Yes	No	Yes	Yes
3b	Development	Specify the objectives, including whether the study describes the development or validation of the model, or both	Yes	Yes	Yes	Yes	No	Yes	Yes	Yes	Yes	Yes	Yes	Yes	Yes	Yes	Yes	Yes	Yes	Yes
4a	Development	Describe the study design or source of data (for example, randomized trial, cohort, or registry data), separately for the development and validation data sets, if applicable	Yes	Yes	Yes	Yes	Yes	Yes	Yes	Yes	Yes	Yes	Yes	Yes	Yes	Yes	Yes	Yes	Yes	Yes
4b	Development	Specify the key study dates, including start of accrual; end of accrual; and, if applicable, end of follow‐up	No	Yes	Yes	Yes	Yes	Yes	Yes	Yes	Yes	Yes	Yes	Yes	Yes	Yes	Yes	No	Yes	Yes
5a	Development	Specify key elements of the study setting (for example, primary care, secondary care, general population) including number and location of centres	Yes	Yes	Yes	Yes	Yes	Yes	Yes	Yes	Yes	Yes	Yes	Yes	Yes	Yes	Yes	Yes	Yes	Yes
5b	Development	Describe eligibility criteria for participants	Yes	Yes	Yes	Yes	Yes	Yes	Yes	Yes	Yes	Yes	Yes	Yes	Yes	Yes	Yes	No	Yes	Yes
5c	Development	Give details of treatments received, if relevant	No	No	Yes	No	Yes	No	Yes	No	Yes	Yes	No	Yes	Yes	Yes	No	No	No	No
6a	Development	Clearly define the outcome that is predicted by the prediction model, including how and when assessed	Yes	Yes	Yes	Yes	Yes	Yes	Yes	Yes	Yes	Yes	Yes	Yes	Yes	Yes	Yes	Yes	Yes	Yes
6b	Development	Report any actions to blind assessment of the outcome to be predicted	No	No	Yes	No	No	No	No	No	No	No	No	Yes	No	No	Yes	No	No	No
7a	Development	Clearly define all predictors used in developing the multivariable prediction model, including how and when they were measured	Yes	Yes	Yes	Yes	Yes	Yes	Yes	Yes	Yes	Yes	Yes	Yes	Yes	Yes	Yes	Yes	Yes	Yes
7b	Development	Report any actions to blind assessment of predictors for the outcome and other predictors	No	No	Yes	No	No	No	No	No	No	No	No	Yes	No	No	Yes	No	No	No
8	Development	Explain how the study size was arrived at.	No	Yes	No	Yes	Yes	Yes	Yes	Yes	Yes	Yes	Yes	Yes	Yes	Yes	Yes	No	Yes	Yes
9	Development	Describe how missing data were handled (for example, complete‐case analysis, single imputation, multiple imputation) with details of any imputation method	No	No	Yes	No	No	No	Yes	No	No	No	No	Yes	No	Yes	Yes	No	No	No
10a	Development	Describe how predictors were handled in the analyses	Yes	Yes	Yes	Yes	Yes	Yes	Yes	Yes	Yes	Yes	Yes	Yes	Yes	Yes	Yes	Yes	Yes	Yes
10b	Development	Specify type of model, all model‐building procedures (including any predictor selection), and method for internal validation	No	Yes	Yes	Yes	Yes	Yes	Yes	Yes	Yes	Yes	Yes	Yes	Yes	Yes	Yes	Yes	Yes	Yes
10c	Validation	For validation, describe how the predictions were calculated	Yes	Yes	Yes	Yes	Yes	Yes	Yes	Yes	Yes	Yes	Yes	Yes	Yes	Yes	Yes	Yes	Yes	Yes
10d	Development	Specify all measures used to assess model performance and, if relevant, to compare multiple models	No	Yes	Yes	Yes	Yes	Yes	Yes	Yes	Yes	Yes	Yes	Yes	Yes	Yes	Yes	Yes	Yes	Yes
10e	Validation	Describe any model updating (for example, recalibration) arising from the validation, if done	No	No	Yes	No	No	No	Yes	Yes	Yes	Yes	No	Yes	Yes	No	No	No	No	No
11	Development	Provide details on how risk groups were created, if done	NA^b^	Yes	NA	NA	NA	Yes	NA	NA	NA	Yes	NA	NA	NA	NA	NA	Yes	Yes	Yes
12	Validation	For validation, identify any differences from the development data in setting, eligibility criteria, outcome, and predictors	No	Yes	Yes	Yes	Yes	Yes	Yes	Yes	Yes	Yes	No	Yes	No	Yes	Yes	No	Yes	Yes
13a	Development	Describe the flow of participants through the study, including the number of participants with and without the outcome and, if applicable, a summary of the follow‐up time. A diagram may be helpful	No	Yes	Yes	Yes	Yes	Yes	No	Yes	No	Yes	No	Yes	No	Yes	Yes	No	Yes	Yes
13b	Development	Describe the characteristics of the participants (basic demographics, clinical features, available predictors), including the number of participants with missing data for predictors and outcome	No	Yes	Yes	Yes	Yes	Yes	No	Yes	Yes	Yes	Yes	Yes	No	Yes	Yes	No	Yes	Yes
13c	Validation	For validation, show a comparison with the development data of the distribution of important variables (demographics, predictors and outcome).	No	Yes	Yes	No	Yes	Yes	Yes	Yes	Yes	Yes	No	Yes	No	Yes	Yes	No	Yes	Yes
14a	Development	Specify the number of participants and outcome events in each analysis	Yes	Yes	Yes	Yes	Yes	Yes	Yes	Yes	Yes	Yes	Yes	Yes	Yes	Yes	Yes	Yes	Yes	Yes
14b	Development	If done, report the unadjusted association between each candidate predictor and outcome	No	Yes	Yes	Yes	Yes	Yes	Yes	Yes	Yes	Yes	Yes	Yes	Yes	Yes	Yes	Yes	Yes	Yes
15a	Development	Present the full prediction model to allow predictions for individuals (that is, all regression coefficients, and model intercept or baseline survival at a given time point)	No	Yes	Yes	Yes	Yes	Yes	Yes	Yes	Yes	Yes	Yes	Yes	Yes	Yes	Yes	Yes	Yes	Yes
15b	Development	Explain how to use the prediction model	No	Yes	Yes	Yes	Yes	Yes	Yes	Yes	Yes	Yes	Yes	Yes	Yes	Yes	Yes	Yes	Yes	Yes
16	Development	Report performance measures (with CIs) for the prediction model	No	Yes	Yes	Yes	Yes	Yes	Yes	Yes	Yes	Yes	Yes	Yes	Yes	Yes	Yes	Yes	Yes	Yes
17	Validation	If done, report the results from any model updating (that is, model specification, model performance)	NA	NA	NA	Yes	NA	NA	NA	NA	Yes	Yes	NA	Yes	Yes	NA	NA	NA	NA	NA
18	Development	Discuss any limitations of the study (such as nonrepresentative sample, few events per predictor, missing data)	Yes	Yes	Yes	No	Yes	Yes	Yes	Yes	Yes	No	Yes	Yes	Yes	No	Yes	No	No	Yes
19a	Validation	For validation, discuss the results with reference to performance in the development data, and any other validation data	No	Yes	Yes	No	Yes	Yes	Yes	Yes	Yes	Yes	No	Yes	Yes	Yes	Yes	Yes	Yes	Yes
19b	Development	Give an overall interpretation of the results, considering objectives, limitations, results from similar studies, and other relevant evidence	Yes	Yes	Yes	Yes	Yes	Yes	Yes	Yes	Yes	Yes	Yes	Yes	Yes	Yes	Yes	Yes	Yes	Yes
20	Development	Discuss the potential clinical use of the model and implications for future research	Yes	Yes	Yes	Yes	Yes	Yes	Yes	Yes	Yes	Yes	Yes	Yes	Yes	Yes	Yes	Yes	Yes	Yes
21	Development	Provide information about the availability of supplementary resources, such as study protocol, Web calculator, and data sets	No	Yes	Yes	No	Yes	Yes	No	Yes	Yes	Yes	No	Yes	Yes	Yes	No	Yes	No	Yes
22	Development	Give the source of funding and the role of the funders for the present study	No	Yes	Yes	No	Yes	Yes	Yes	Yes	Yes	Yes	Yes	Yes	Yes	Yes	Yes	Yes	Yes	Yes

Note

a: Transparent Reporting of a multivariable prediction model for Individual Prognosis Or Diagnosis. b: Not applicable.

Altogether, 18 eligible datasets were selected (GSE70425 included two different miRNA expression matrixes based on different cell types). MiRNA expression data were obtained from 14 datasets, whereas snoRNA expression data were obtained from one dataset. One dataset provided lncRNA expression data, and three datasets offered circRNA‐related data (Table 3).

**TABLE 3:** The characteristics of microarray and sequencing dataset

Dataset ID	Platform	Detection method	Participants region	Mean age (years)	HIV^+^ number	Treatment status	Diagnosis method	Group setting	Sample type	NcRNA^a^ type
GSE131174	GPL16791	Sequencing	South America	>18	NA^b^	Some	Comprehensive diagnosis	TB^c^, untreated LTBI^d^, treated LTBI and HC^e^	WB^f^	MiRNA^g^ and snoRNA^h^
GSE34608	GPL7731	Microarray	Central Europe	>18	0	None	NA	TB, sarcoidosis and HC	WB	MiRNA
GSE39163	GPL7731	Microarray	Mixed Europe	NA	NA	NA	Aetiological evidence, radiology and TST^i^	TB and LTBI	WB	MiRNA
GSE119494	GPL11154	Sequencing	East Asia	>18	0	NA	Symptom, radiology and culture	TB and HC	PBMC^j^	MiRNA
GSE131708	GPL23365	Microarray	East Asia	≥18	0	NA	Culture, smear or Xpert	TB, viral meningitis and HC	PBMC	MiRNA
GSE15977	GPL8227	Microarray	East Asia	NA	NA	NA	NA	TB, LTBI and HC	PBMC	MiRNA
GSE25435	GPL10850	Microarray	East Asia	NA	NA	NA	NA	TB, LTBI and HC	PBMC	MiRNA
GSE29190	GPL10850	Microarray	East Asia	≥18	0	None	Symptom, smear and radiology	TB, LTBI and HC	PBMC	MiRNA
GSE70425	GPL15159	Microarray	South Africa	>18	0	None	Symptom, radiology and culture	TB and LTBI	Monocytes Granulocytes	MiRNA
SRP029907*	/	Sequencing	East Asia	>18	0	None	Symptom, culture and radiology	TB and LTBI	Serum	MiRNA
SRP032650*	/	Sequencing	NA	NA	NA	NA	NA	TB, LTBI, HC with BCG^k^ and HC without BCG	Serum	MiRNA
GSE116542	GPL19117	Microarray	East Asia	>18	0	None	Culture	TB and HC	Exosome	MiRNA
GSE124120	GPL16791	Sequencing	East Asia	≥18	0	NA	Culture and smear	TB, LTBI and HC	Exosome	MiRNA
GSE101805	GPL16956	Microarray	East Asia	NA	0	NA	Culture and smear	TB, pneumonia and HC	Plasma	LncRNA^l^
GSE103188	GPL23259	Microarray	East Asia	>18	0	NA	Comprehensive diagnosis	TB and HC	PBMC	CircRNA^m^
GSE117563	GPL21825	Microarray	East Asia	>18	0	None	Symptom and culture or smear	TB and HC	PBMC	CircRNA
GSE106953	GPL21825	Microarray	East Asia	>18	0	None	Symptom and culture or smear	TB and HC	Plasma	CircRNA

Note

a: non‐coding RNA; b: non‐available; c: tuberculosis; d: latent tuberculosis infection; e: healthy control; f: whole blood; g: microRNA; h: small nucleolar RNA; i: tuberculin skin test; j: peripheral blood mononuclear cell; k: bacillus Calmette‐Guérin; l: long non‐coding RNA; m: circular RNA.

* These 2 datasets were stored in Sequence Read Archive (SRA) database.

### 3.2 The performance of each ncRNA panel in different datasets

#### 3.2.1 MiRNA diagnostic panels

Of all miRNA diagnostic models implemented on available validation datasets, Cui 2017 harboured the highest specificity at 90% sensitivity (88%) and AUC (89%, 95% CI: 0.86‐0.92), followed by Latorre 2015 (specificity at 90% sensitivity: 83%, AUC: 86%, 95% CI: 0.86‐0.88). Low heterogeneity was identified in the pooled results of these two models (Table 4). In seven aetiological‐confirmation validation datasets, the specificity of Cui 2017 at 90% sensitivity increased to 92% and AUC climbed to 95% (95% CI: 0.93‐0.97). The *I* ^2^ of Cui 2017 in these seven datasets declined to 0%. However, Latorre 2015 had a declined specificity at 90% sensitivity of 11% and AUC of 68% (95% CI: 0.64‐0.72) in six aetiological‐confirmation validation datasets (Table 4).

**TABLE 4:** The performance of 13 miRNA models in all available miRNA datasets

Model Name	Validation set number	Chi‐square	*P* of Chi‐square	*I* ^2 a^	AUC^b^ (95% CI^c^)	Specificity at 90% sensitivity	Sensitivity (95% CI)	Specificity (95% CI)	PPV^d^ (95% CI)	NPV^e^ (95% CI)
Latorre 2015	10	0.155	.463	0%	86% (0.82‐0.88)	83%	41% (0.27‐0.56)	86% (0.78‐0.92)	64% (0.48‐0.78)	70% (0.58‐0.79)
Pan 2019	11	1.957	.188	0%	59% (0.54‐0.63)	45%	56% (0.34‐0.76)	58% (0.47‐0.68)	43% (0.32‐0.54)	68% (0.45‐0.85)
Wang 2011	8	2.471	.145	19%	46% (0.41‐0.50)	1%	31% (0.17‐0.51)	76% (0.51‐0.91)	52% (0.28‐0.76)	58% (0.46‐0.70)
Zhou 2016	10	0.000	.500	0%	51% (0.47‐0.55)	10%	53% (0.39‐0.66)	48% (0.37‐0.61)	45% (0.33‐0.58)	56% (0.43‐0.69)
Barry 2018	11	3.622	.082	45%	52% (0.47‐0.56)	18%	94% (0.76‐0.99)	13% (0.06‐0.26)	37% (0.27‐0.48)	70% (0.50‐0.84)
Cui 2017	11	3.221	.100	38%	89% (0.86‐0.92)	88%	8% (0.01‐0.40）	91% (0.84‐0.95)	49% (0.27‐0.71)	68% (0.56‐0.79)
Duffy 2018	0	NA^f^	NA	NA	NA	NA	NA	NA	NA	NA
Miotto 2013‐RVM	9	11.757	.001	83%	60% (0.56‐0.64)	4%	98% (0.62‐1.00)	0% (0.00‐0.97)	40% (0.32‐0.50)	58% (0.36‐0.77)
Miotto 2013‐AIC logistic regression	9	4.165	.062	52%	43% (0.39‐0.48)	35%	58% (0.27‐0.83)	42% (0.31‐0.55)	35% (0.23‐0.49)	62% (0.37‐0.82)
Qi 2012	10	5.495	.032	64%	77% (0.73‐0.81)	0%	92% (0.76‐0.98)	0% (0.00‐1.00)	38% (0.30‐0.47)	31% (0.18‐0.47)
Zhang 2013	5	10.487	.003	81%	51% (0.46‐0.71)	17%	10% (0.01‐0.55)	84% (0.51‐0.96)	29% (0.13‐0.54)	57% (0.44‐0.70)
Alipoor 2019	11	2.091	.176	4%	67% (0.63‐0.71)	53%	17% (0.05‐0.45)	77% (0.66‐0.85)	32% (0.12‐0.63)	66% (0.55‐0.75)
Hu 2019	0	NA	NA	NA	NA	NA	NA	NA	NA	NA

Note

a: inconsistence index; b: area under curve; c: 95% confidence interval; d: positive predictive value; e: negative predictive value; f: non‐available.

#### 3.2.2 Other types of ncRNA diagnostic panels

No validation sets were accessed for one miRNA and snoRNA model, lncRNA model and circRNA model. Two validation sets were accessed for three circRNA models, which were not sufficient to pool. The performance of these models in training set is shown in Table S1.

### 3.3 Meta‐regression

We performed meta‐regressions for 13 models implemented on 12 miRNA panels (Table 5), whereas we cannot conduct further meta‐regressions regarding limited available lncRNA, circRNA and snoRNA.

**TABLE 5:** The results of meta‐regression in 13 miRNA diagnostic models

Model Name	Covariate (Consistency between training set * and validation set)	Category	Validation set number	Sensitivity (95% CI^a^)	P1^†^	Specificity (95% CI)	P2^‡^
Latorre 2015	Sample type	Consistency	9	37% (0.24‐0.51)	.18	88% (0.82‐0.94)	.18
		Inconsistency	1	64% (0.35‐0.92)		63% (0.29‐0.96)	
	Ethnicity	Consistency	6	47% (0.25‐0.68)	.45	87% (0.79‐0.95)	.25
		Inconsistency	4	37% (0.20‐0.54)		85% (0.74‐0.96)	
Pan 2019	Sample type	Consistency	9	58% (0.33‐0.83)	.59	61% (0.51‐0.72)	.10
		Inconsistency	2	43% (0.00‐0.98)		28% (0.00‐0.57)	
	Ethnicity	Consistency	6	66% (0.37‐0.94)	.44	62% (0.44‐0.80)	.96
		Inconsistency	5	48% (0.22‐0.73)		55% (0.42‐0.69)	
Wang 2011	Sample type	Consistency	7	27% (0.13‐0.41)	.21	76% (0.54‐0.98)	.68
		Inconsistency	1	55% (0.24‐0.85)		77% (0.25‐1.00)	
	Ethnicity	Consistency	4	52% (0.30‐0.75)	.04	73% (0.42‐1.00)	.92
		Inconsistency	4	22% (0.08‐0.36)		78% (0.52‐1.00)	
Zhou 2016	Sample type	Consistency	8	54% (0.38‐0.69)	.88	52% (0.39‐0.65)	.22
		Inconsistency	2	50% (0.22‐0.78)		30% (0.02‐0.58)	
	Ethnicity	Consistency	8	49% (0.32‐0.65)	.38	42% (0.28‐0.56)	.10
		Inconsistency	2	62% (0.39‐0.86)		69% (0.46‐0.91)	
Barry 2018	Sample type	Consistency	1	100% (1.00‐1.00)	NA	0% (0.00‐0.00)	NA
		Inconsistency	10	93% (0.84‐1.00)		15% (0.06‐0.23)	
	Ethnicity	Consistency	6	90% (0.71‐1.00)	.93	15% (0.00‐0.31)	.51
		Inconsistency	5	96% (0.88‐1.00)		11% (0.00‐0.22)	
Cui 2017	Sample type	Consistency	1	0% (0.00‐0.00)	NA	100% (1.00‐1.00)	NA
		Inconsistency	10	9% (0.01‐0.41)		91% (0.84‐0.95)	
	Ethnicity	Consistency	6	18% (0.00‐0.54)	.82	97% (0.91‐1.00)	.11
		Inconsistency	5	5% (0.00‐0.16)		89% (0.82‐0.97)	
Duffy 2018	Sample type	Consistency	0	NA^b^	NA	NA	NA
		Inconsistency	0	NA		NA	
	Ethnicity	Consistency	0	NA	NA	NA	NA
		Inconsistency	0	NA		NA	
Miotto 2013‐RVM	Sample type	Consistency	0	NA	NA	NA	NA
		Inconsistency	9	98% (0.62‐1.00)		0% (0.00‐0.97)	
	Ethnicity	Consistency	5	88% (0.62‐1.00)	.05	5% (0.00‐0.22)	.15
		Inconsistency	4	100% (1.00‐1.00)		0% (0.00‐0.02)	
Miotto 2013‐AIC logistic regression	Sample type	Consistency	0	NA	NA	NA	NA
		Inconsistency	9	58% (0.27‐0.83)		42% (0.31‐0.55)	
	Ethnicity	Consistency	5	87% (0.69‐1.00)	<.01	50% (0.32‐0.68)	.21
		Inconsistency	4	28% (0.13‐0.44)		35% (0.20‐0.50)	
Qi 2012	Sample type	Consistency	1	0% (0.00‐0.00)	NA	0% (0.00‐0.00)	1.00
		Inconsistency	9	91% (0.91‐0.91)		0% (0.00‐0.00)	
	Ethnicity	Consistency	6	82% (0.59‐1.00）	.53	0% (0.00‐0.00)	1.00
		Inconsistency	4	95% (0.84‐1.00）		0% (0.00‐0.00)	
Zhang 2013	Sample type	Consistency	0	NA	NA	NA	NA
		Inconsistency	5	10% (0.01‐0.55)		84% (0.51‐0.96)	
	Ethnicity	Consistency	3	13% (0.00‐0.56)	.56	63% (0.38‐0.88)	.77
		Inconsistency	2	3% (0.00‐0.14)		100% (1.00‐1.00)	
Alipoor 2019	Sample type	Consistency	1	0% (0.00‐0.00)	NA	100% (1.00‐1.00)	NA
		Inconsistency	10	17% (0.05‐0.46)		76% (0.65‐0.85)	
	Ethnicity	Consistency	6	15% (0.00‐0.42)	.51	74% (0.57‐0.91)	.24
		Inconsistency	5	18% (0.00‐0.43)		78% (0.66‐0.90)	
Hu 2019	Sample type	Consistency	NA	NA	NA	NA	NA
		Inconsistency	NA	NA		NA	
	Ethnicity	Consistency	NA	NA	NA	NA	NA
		Inconsistency	NA	NA		NA	

Note

a: confidence interval; b: non‐available.

* The training set referred to the dataset used to train the model in this work and the detailed information of training set of each model was provided in Table S1.

^†^ P1 referred to the *P* value when comparing the sensitivity of each model in consistent subgroup and inconsistent subgroup.

^‡^ P2 referred to the *P* value when comparing the specificity of each model in consistent subgroup and inconsistent subgroup.

#### 3.3.1 Sample type

NcRNA expression profiles in WB, PBMC or other blood cells (non‐cell‐free samples) were different from these in serum, plasma or exosome (cell‐free samples).^39^ Herein, we performed meta‐regression based on the consistency of sample type between training set and validation sets.

For miRNA panels based on non‐cell‐free samples, Pan 2019 and Latorre 2015 showed the highest sensitivity (58%) and specificity (88%) in consistent subgroup. For miRNA panels based on cell‐free samples, Miotto 2013‐RVM and Cui 2017 harboured the highest sensitivity (98%) and specificity (91%) in inconsistent subgroup.

#### 3.3.2 Ethnicity

Ethnicity was considered as a covariate based on the country of training sets of each model (African, Caucasian and Asian), regarding the reported differences in miRNA expression among different ethnicities in TB.^40^

In the consistent subgroup, Barry 2018 showed highest sensitivity (90%), whereas Cui 2017 still had the highest specificity (97%). In another subgroup, Miotto 2013‐RVM harboured the highest sensitivity of 100%, and still, Cui 2017 showed the highest specificity of 89%. Significant improvement of sensitivity was found in Miotto 2013‐AIC logistic regression (*P* < .01), and marginally significance was identified in sensitivity of Miotto 2013‐RVM (*P* = .05). Significant difference was also observed between these two subgroups regarding sensitivity of Wang 2011 (*P* = .04).

### 3.4 Biological function of ncRNAs in panels

#### 3.4.1 MiRNAs in related panels

**FIGURE 2:**
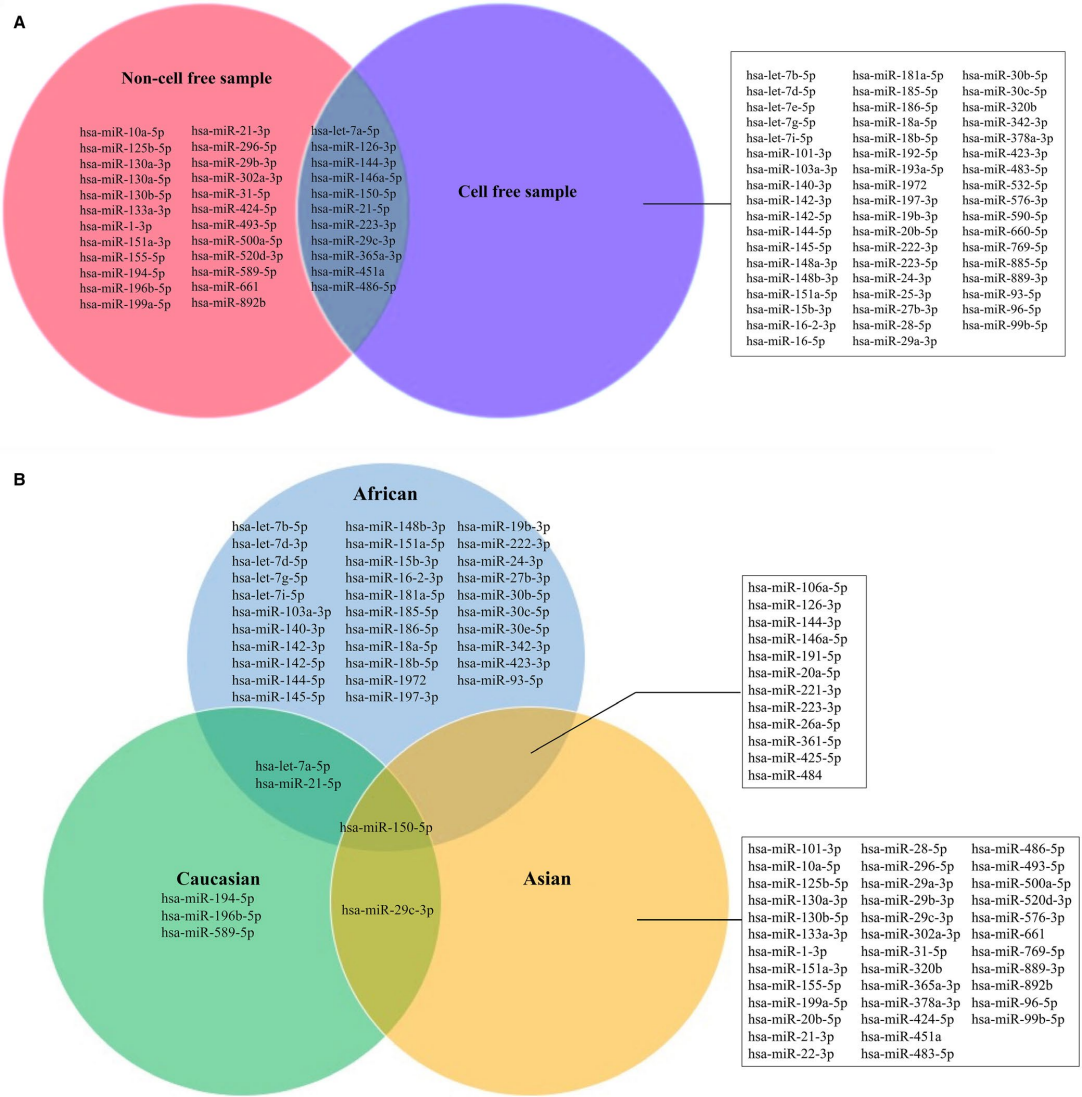
The Venn diagram of distribution of miRNAs in included panels. A, The distribution of miRNAs in included panels which were built based on different sample types; B, The distribution of miRNAs in included panels which were built based on different ethnicities. PBMC: peripheral blood mononuclear cells; WB, whole blood

Considering that samples of Miotto 2013 came from two different continents, further analyses for miRNAs in this panel were not performed. The inclusion of all miRNAs in the remaining 12 related panels was summarized according to ethnicity and sample type in their original papers (Figure 2), and miR‐150‐5p was selected for panels based on different ethnicities and sample types.

**FIGURE 3:**
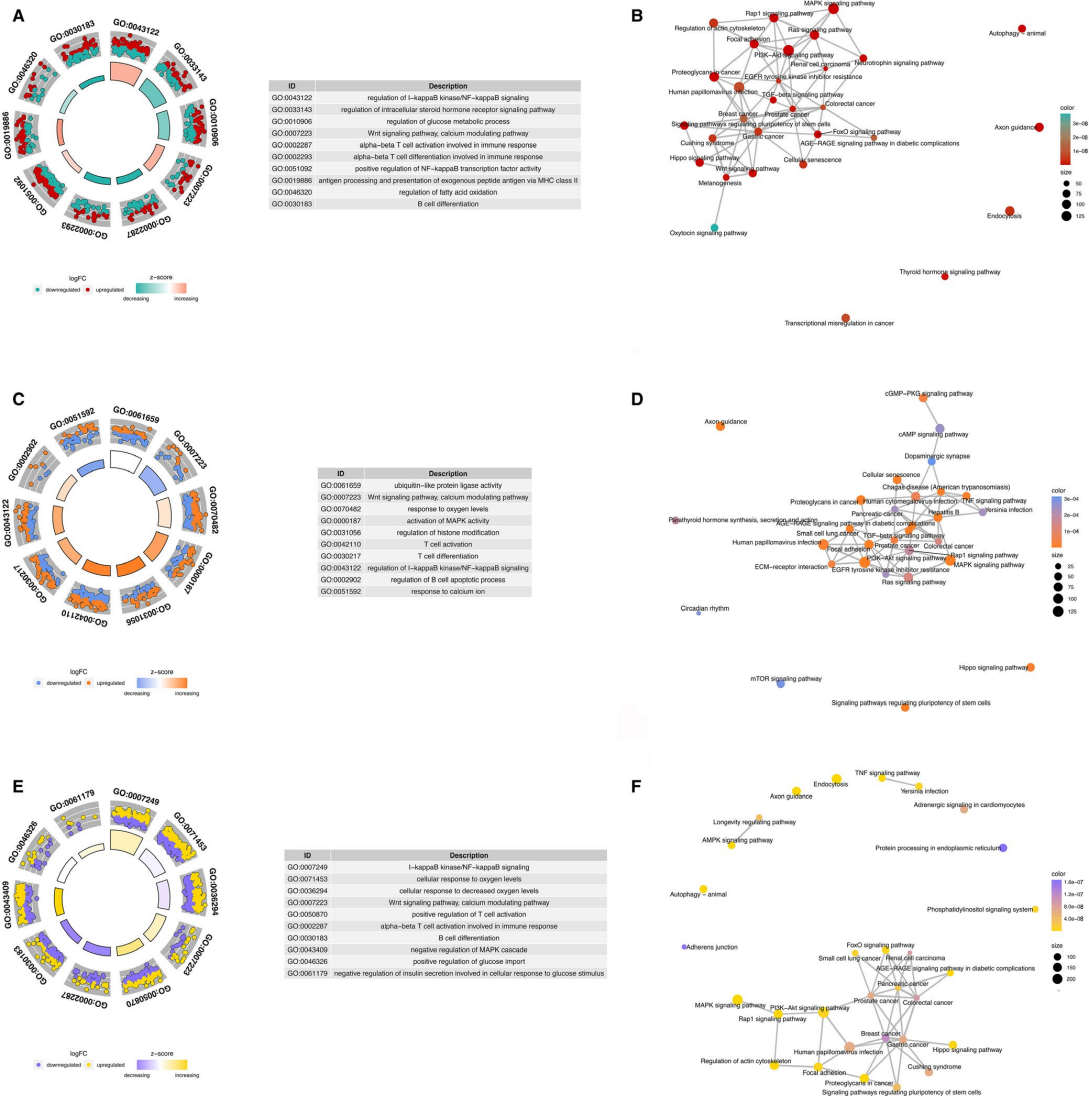
GO and KEGG analysis of targeted genes of miRNAs in included panels based on different ethnicities (different country where study was conducted, grouped into regions). In circular plot, the inner circle shows *z*‐score which indicated whether the biological process is more likely to be decreased (negative value) or increased (positive value), whereas the outer circle shows the GO ID and the distribution of up and down‐regulated genes. In emapplot, each node represents a pathway of enrichment and top 30 pathways of enrichment in KEGG analysis are drawn. The node size corresponds to the number of different genes enriched under the pathway, and the colour of the node corresponds to the value of *P*.adjust, from small to large, corresponding to different colours. A, Circular plot of GO analysis for miRNAs in included panels which were built based on African race; B, Emapplot of KEGG analysis for targeted genes of miRNAs in included panels which were built based on African ethnicity; C, circular plot of GO analysis for targeted genes of miRNAs in included panels which were built based on Caucasian ethnicity; D, Emapplot of KEGG analysis for targeted genes of miRNAs in included panels which were built based on Caucasus; E: circular plot of GO analysis for targeted genes of miRNAs in included panels which were built based on Asian race; F, Emapplot of KEGG analysis for targeted genes of miRNAs in included panels which were built based on Asian race

**FIGURE 4:**
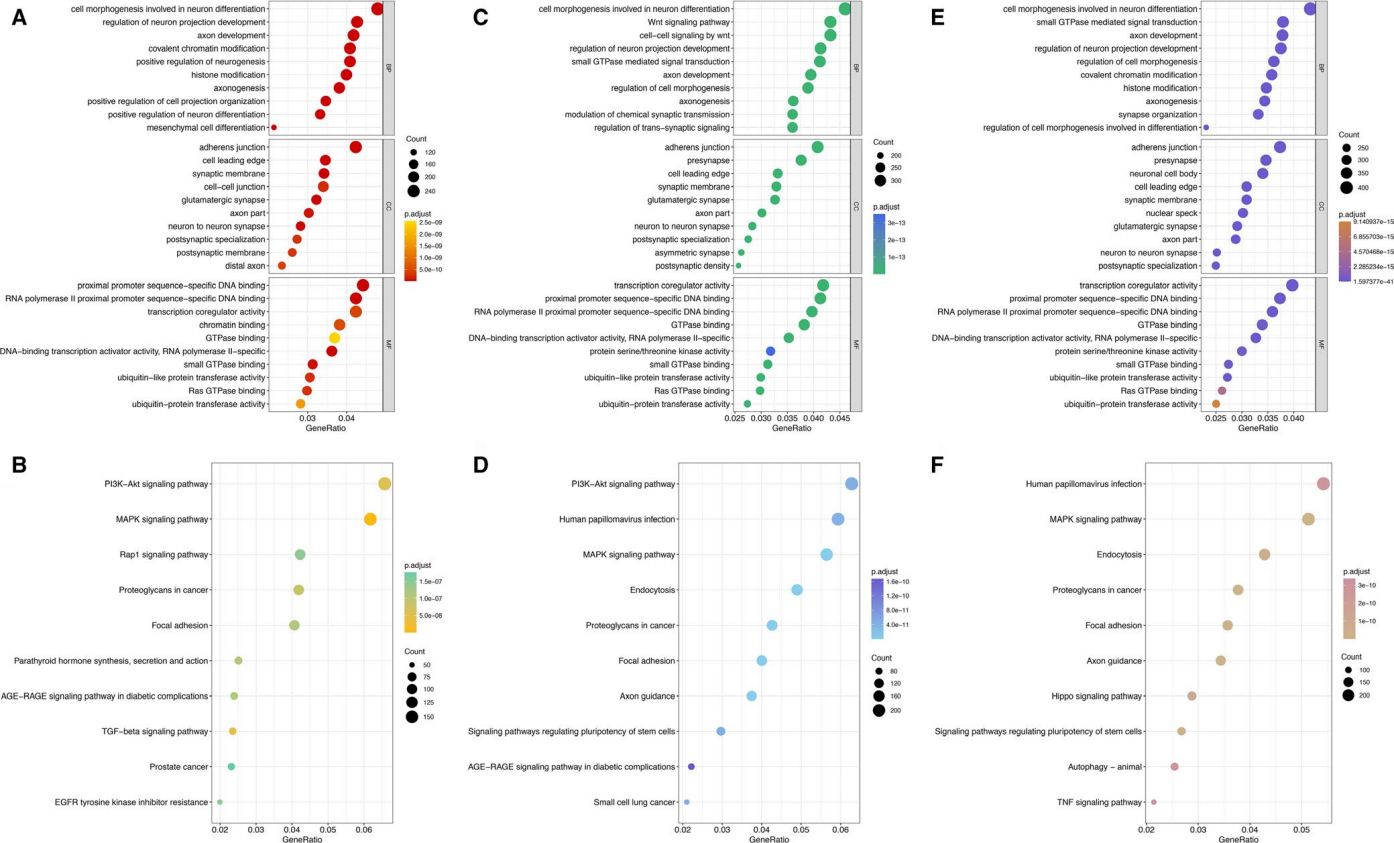
GO and KEGG analysis of targeted genes of miRNAs in included panels based on different sample types. In dotplot, abscissa axis is GeneRatio which represents the ratio of the number of differentially expressed genes under the pathway to the total number of differentially expressed genes. The vertical axis is the description information of the enriched pathways. The top 10 pathways of enrichment are shown. The colour of the dot in the graph corresponds to the value of *P*.adjust. The size of the dot corresponds to the number of differently expressed genes under specific GO terms. A, Dotplot of GO analysis for targeted genes of miRNAs in both panels using non‐cell‐free samples and cell‐free samples; B, Dotplot of KEGG analysis for targeted genes of miRNAs in both panels using non‐cell‐free samples and cell‐free samples; C, Dotplot of GO analysis for targeted genes of miRNAs in included panels using non‐cell‐free samples; D, Dotplot of KEGG analysis for targeted genes of miRNAs in included panels using non‐cell‐free samples; E, Dotplot of GO analysis for targeted genes of miRNAs in included panels using cell‐free samples; F, Dotplot of KEGG analysis for targeted genes of miRNAs in included panels using cell‐free samples. BP, biological process; CC, cellular component; MF, molecular function

GO analysis indicated that target genes of miRNAs undertook the function of immune cell activation and oxidative stress, whereas KEGG analysis implied that these targeted genes involved in Wnt signalling pathway, MAPK signalling pathway, PI3K‐Akt signalling and some infections including influenza A and hepatitis B. The results of GO and KEGG analysis for miRNAs in panels based on different ethnicities and samples are present in Figures 3 and 4, respectively.

#### 3.4.2 LncRNAs in related panels

GO analysis showed that targeted genes of lncRNAs were related to divalent inorganic cation transmembrane transporter activity, whereas KEGG analysis indicated that these genes mainly involved in the process of ferroptosis.

#### 3.4.3 CircRNAs in related panels

**FIGURE 5:**
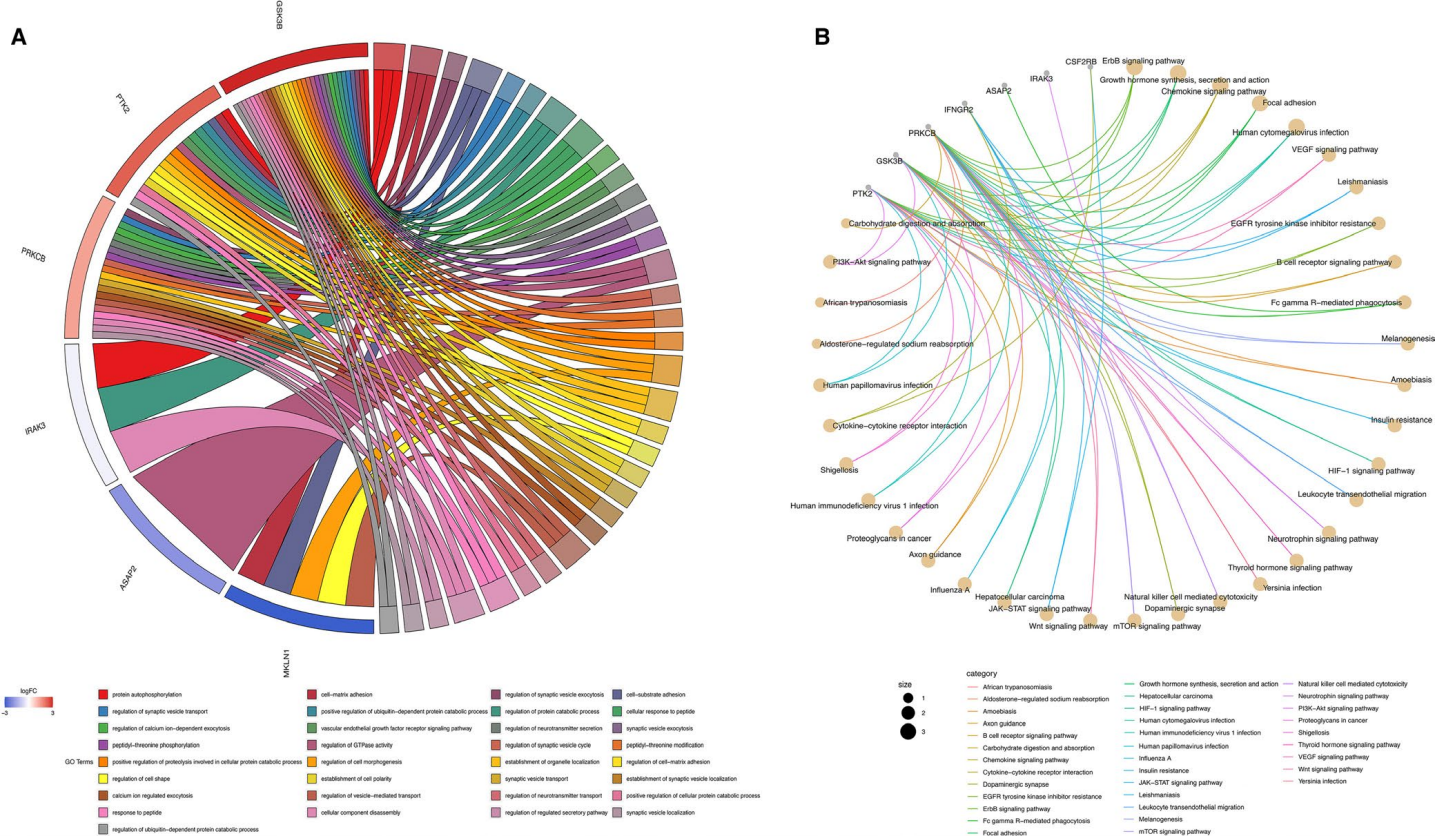
GO and KEGG analysis of parental genes of circRNAs in included panels. In chord plot, up‐ or down‐regulated genes are shown in the left side, whereas GO terms are listed on the right side. The ribbons represent a certain gene in a certain GO term, and the thickness of the ribbons is proportional to the number of parental genes. In cnetplot, the grey dots represent parental genes and the yellow dots represent enriched pathways. The top 35 enriched pathway in KEGG analysis was shown. The size of pathways nodes corresponds to the number of genes enriched. A, The chord plot of GO analysis for parental genes of circRNAs in included panels; B, The cnetplot of KEGG analysis for parental genes of circRNAs in included panels

GO analysis showed that the parental genes of circRNA participated in GTPase activity, protein autophosphorylation and other biological processes. KEGG suggested that these parental genes participated in many important pathways including Wnt signalling pathway and JAK‐STAT signalling pathway and some infection such as influenza A, HIV, cytomegalovirus and papillomavirus (Figure 5).

## 4 DISCUSSION

In this work, 19 ncRNA diagnostic models (from 18 ncRNA diagnostic panels) were rebuilt and assessed in 18 ncRNA datasets. Among all models, the specificity at 90% sensitivity and AUC of Cui 2017 reached to 88% and 89% (95% CI: 0.86‐0.92) in all available validation sets, whereas they were 90% and 95% (95% CI: 0.93‐0.97) in seven aetiological‐confirmation validation datasets. Latorre 2015 also showed high specificity at 90% sensitivity and AUC in all available validation sets, but not in six aetiological‐confirmation validation datasets. Meta‐regression indicated that both sample types and ethnicity were confounding factors and different models reacted differently to these factors. In addition, potential biofunctions of ncRNAs in panels were also predicted to explore the maximum value of these TB‐related ncRNAs.

Among all 18 ncRNA diagnostic panels, both Latorre 2015 and Cui 2017 showed excellent performance in validation datasets. MiRNAs in these two panels were selected from relatively large discovery cohorts. The discovery cohort of Latorre 2015 included 17 TB patients, 17 LTBI and 16 HCs, whereas 50 TB patients and 31 HCs were recruited into the discovery cohort of Cui 2017. Relatively, large discovery cohorts can ensure the efficiency of differential analysis, whereas the results of relatively small cohorts may be influenced by individual heterogeneity. These differently expressed miRNAs in these two panels were discovered by microarray or sequencing. Both microarray and sequencing have large coverages and can fully explore TB‐related specific markers, whereas PCR does not. In further validation, only Cui 2017 fulfil the criteria of the target product profile (TPP) provided by WHO.^4^ Relatively, few miRNAs included in this panel (three miRNAs) and stable performance in different ethnicities further facilitate its clinical application. Three miRNAs in Cui 2017 were chosen by the cross‐validation of different groups (HCs, cavitary TB and non‐cavitary TB). During selection, the diagnostic performance of each single miRNA was also considered, to ensure overall discriminatory effectiveness between TB and HC. Linear combination used in Cui 2017 took into account both the miRNA expression of each dataset itself and the weight of included variables, which might confer its excellent generalization ability. However, a miRNA extensively participates in various biological and physiological pathways and thus has potential to be a biomarker for several different diseases, such as miR769_5p, miR320a and miR22_3p in Cui 2017.^41^, ^42^, ^43^ Multiple roles of miRNAs in biological activities can decrease their specificity in a given disease. Combining several differently expressed miRNAs in a specific disease into one panel is a common method to improve the diagnostic specificity. It still needs more comprehensive validation in diverse populations to ensure the performance of panels before clinical application. For Cui 2017, the capacity of this panel in different clinical sample types, predicting the progress of TB and diagnosing paediatric TB still needs further exploration.

For other models with unsatisfactory performance, failure to reproduce these models, a methodological difference and heterogeneity across different datasets may contribute to this result.^44^ In this paper, we developed models as close as possible to original papers and thought that the impact of such alterations on the model can be negligible, which is also supported by other scholars.^17^ Now, numerous models have been proliferated, whereas most of them do not provide detailed parameters of modelling which is of utmost importance for the performance of models.^45^ Missing parameters impede model reproduction, external validation and further improvement. Therefore, we suggest that the details of modelling including parameters, algorithms composition and even the all coefficients should be provided. Some algorithm cheat sheets have been plotted to assist scholars to identify an algorithm for their own data.^46^, ^47^ Briefly, identifying research purpose, the characteristics of raw data and requirements for algorithm features can guide the choice of modelling methods.^46^ Heterogeneity across different datasets is also a cause of fluctuations in the performance of panels.^48^ Usually, the diagnostic ability of panels would decrease in diverse settings where factors are different from those participating in the model derivation. Siontis et al^49^ reported that only 1/118 model exhibited excellent AUC in different settings, and unsatisfactory results also appear in other systematic review.^50^ Constructing a model is a multiphase process, and during this process, optimal algorithm and parameters for specific training set can be found; however, this does not mean that the model performs equally well in other datasets. Herein, ensuring the rigour of the entire modelling process determines the performance and generalization capabilities of models.

Researchers have put forward various approaches to comprehensively evaluate model quality. In 2014, Steyerberg et al^51^ proposed seven steps for development and an ABCD for validation. In 2015, Collins et al^18^ developed the statement of TRIPOD. In this article, we further assessed the whole modelling process according to TRIPOD and found only Hu et al^3^ met all the criteria and thus how to ensure the standardization of modelling while paying attention to the performance of model deserves to be valued.

Meta‐regression indicated that the influence of ncRNA sources (sample types) and targeted populations could not be neglected. For instance, Miotto 2013‐AIC regression and Wang 2011 had AUC < 50% in all available datasets, which might be caused by the different directions of the relationship between TB risk and predictive scores of models. In the training set, subjects with higher predictive scores were more likely to be divided into TB group, and there was on the contrary in some validation sets with different ethnic populations from training set. Meta‐regression further confirmed this finding that the performance of these two models was improved in ethnicity‐consistency subgroup. Besides, the Venn diagram of miRNA distribution in this paper (Figure 2) also supported that the expression patterns of ncRNAs were shown to differ based on different sample types and ethnicities. Of note, different models respond differently to sample types and ethnicities. As we discussed before, Miotto 2013‐AIC regression and Wang 2011 presented a certain degree of ethnic specificity, whereas Latorre 2015 and Pan 2019 were insensitive to ethnicities. It is difficult to judge which model is the optimal, the one with better generalization ability or with better diagnostic performance for the specific population, the one with the highest sensitivity or with the highest specificity. We recommend that the models with excellent sensitivity should be selected for high TB burden areas, whereas these with outstanding specificity ought to be applicable to low TB burden regions. Desired sensitivity enables to improve generalizability and capacity of triaging TB patients and also reduces the costs and requirements of using high accurate detection tools,^52^ which is beneficial for ease the pressure in high TB burden areas where are usually economically disadvantaged.^53^ Correctly excluding subjects who do not suffer from TB is a more cost‐effective approach for low TB burden areas; thus, high specificity ensures the reliability of classifying outcome.^52^ In summary, the evaluation of model ought to take study objectives, disease burden and prevalence, and socio‐economic requirements into consideration.^54^

GO analysis and KEGG analysis demonstrated that most ncRNAs in included panels involved into Wnt signalling pathway, oxidative stress and immune cell activation and differentiation, which were closely related to the pathogenesis of TB.^55^, ^56^, ^57^ MiR‐150‐5p was selected by 3/13 miRNA panels which targeted different populations and used different sample types. MiR‐150‐5p is widely expressed in immune cells and is responsible for the development of lymphocytes, lung cancer and acute lung injury.^58^ Through conducting both clinical study and mice experiment, Ghorpade et al^59^ confirmed that miR‐150‐5p could suppress TLR2 responses by targeting an adaptor protein of TLR2 signalling, MyD88, and thus regulate the host‐MTB interactions. Chen et al^60^ implied that miR‐150‐5p interacted with the transcription factor c‐Myb to inhibit memory CD8 T cell development, which played a crucial role in a rapid response to reinfection. In addition, Zhou et al^25^ reported that miR‐150‐5p outperformed any other single miRNA when diagnosing TB. Wang et al^61^ further revealed the value of miR‐150‐5p as a promising marker to differentially diagnose whether a pleural effusion is tuberculous or benign lesion. Clearly, miR‐150‐5p not only can be regarded as a valuable biomarker of TB but also a key molecule in the pathogenesis of TB. Exploring the targeted therapy and diagnostic model of TB based on miR‐150‐5p may yield insights into ameliorate the burden brought by TB.

Although multiple detection technologies have not been applied into clinical practice, simplicity, rapidity and low cost make multiple detection technologies have promising application prospects.^62^, ^63^, ^64^ Once multiple detection technologies enter into clinical practice, ncRNAs in panels can be detected simultaneously and TB risk score can be calculated quickly. It is also noted that the development of loop‐mediated isothermal amplification (LAMP) has accelerated the shift of ncRNA detection pattern from laboratory to point‐of‐care testing (POCT).^65^ LAMP can amplify samples at a fixed temperature, which determines the characteristics of simplicity, rapidity and no high requirements for laboratory environment of these methods. Many studies have shown that LAMP has wide application ranges, especially in limited‐resource regions.^66^, ^67^, ^68^ Benefiting from the rapid development of these technologies, ncRNA can play an increasingly valuable role. Therefore, it is important to systematically assess reported TB‐related ncRNA diagnostic panels and thus offer a certain support for clinical choice in diverse situations. Inevitably, this work suffers from some limitations. Missing parameters and modelling steps prevented us from completely reproducing these models. Moreover, limited available datasets restricted us to further analyse lncRNA, circRNA and snoRNA panels and also have a negative impact on improving the effectiveness of evaluation for miRNA panels. The capacity of these panels in predicting TB progression and diagnosing paediatric TB failed to be assessed due to lacking available datasets. Large‐scale prospective validation in diverse populations is a necessary step for the entry of these panels into the clinic.

## 5 CONCLUSION

Cui 2017 showed strong generalization ability and outperformed in both all available validation sets and aetiological‐confirmed validation sets, in line with the requirements of TPP. Cui 2017 had potential for applying into clinical practice. It is worthy to notice that when applying a model, clinicians should clarify the application scope of this model and calibrate this model to the local situation by re‐evaluating the threshold and/or coefficient of variables, to maximize the diagnostic value of the model.

## CONFLICT OF INTERESTS

The author reports no conflicts of interest in this work.

## AUTHOR CONTRIBUTIONS

**Mengyuan Lyu:** Conceptualization (equal); Data curation (lead); Formal analysis (lead); Investigation (equal); Methodology (equal); Software (equal); Validation (equal); Writing‐original draft (equal); Writing‐review & editing (equal). **Yuhui Cheng:** Formal analysis (equal); Methodology (equal); Writing‐original draft (equal); Writing‐review & editing (equal). **Jian Zhou:** Formal analysis (equal); Methodology (equal); Writing‐original draft (equal); Writing‐review & editing (equal). **Weelic Chong:** Formal analysis (equal); Writing‐original draft (equal); Writing‐review & editing (equal). **Yili Wang:** Formal analysis (equal); Writing‐original draft (equal); Writing‐review & editing (equal). **Wei Xu:** Conceptualization (lead); Methodology (equal); Project administration (equal); Resources (lead); Supervision (equal); Writing‐original draft (equal); Writing‐review & editing (equal). **Binwu Ying:** Conceptualization (lead); Funding acquisition (lead); Project administration (lead); Supervision (lead); Writing‐original draft (lead); Writing‐review & editing (lead).

## Supporting information

Supplementary Material

## Data Availability

Data sharing is not applicable to this article as no new data were created or analysed in this study.
