# Supplementary material for: Systematic evaluation, verification and comparison of tuberculosis‐related non‐coding RNA diagnostic panels
Source: J Cell Mol Med. 2020 Dec 13;25(1):184–202. doi: 10.1111/jcmm.15903 (PMC7810967; doi:10.1111/jcmm.15903)
Supplement: Supplementary file 1 — Supplementary Material [file JCMM-25-184-s001.docx]

| **Model name** | **Reference ^*^** | **Training set** | **Sensitivity** | | **Specificity** | | **Area under curve** | |
| --- | --- | --- | --- | --- | --- | --- | --- | --- |
|  |  |  | **Original model** | **Rebuilt model** | **Original model** | **Rebuilt model** | **Original model** | **Rebuilt model** |
| **Latorre 2015** | 23 | GSE29190 | 91% | 100% | 88% | 100% | NA ^a^ | 100% |
| **Pan 2019** ^†^ | 15 | GSE131708 | 94%/91% | 100% | 71%/87% | 100% | 88%/89% | 100% |
| **Wang 2011** | 24 | GSE29190 | NA | 31% | NA | 100% | NA | 100% |
| **Zhou 2016** | 25 | GSE34608 | 96% | 100% | 100% | 69% | 100% | 100% |
| **Barry 2018** | 26 | GSE116542 | 94% | 91% | 88% | 75% | 98% | 84% |
| **Cui 2017** | 27 | GSE116542 | NA | 64% | NA | 88% | 91% | 76% |
| **Duffy 2018** | 28 | GSE116542 | NA | 100% | NA | 100% | 70% | 100% |
| **Miotto 2013-RVM** | 29 | GSE116542 | 86% | 92% | 79% | 50% | 82% | 64% |
| **Miotto 2013-AIC logistic regression** |  |  | 71% | 100% | 82% | 100% | 95% | 100% |
| **Qi 2012** | 30 | GSE116542 | NA | 82% | NA | 75% | 86% | 80% |
| **Zhang 2013** | 31 | GSE116542 | 95% | 100% | 92% | 100% | 98% | 100% |
| **Alipoor 2019** | 32 | GSE116542 | NA | 55% | NA | 88% | 78% | 60% |
| **Hu 2019 ^‡^** | 3 | GSE116542 | 100%/95% | 91% | 81%/76% | 75% | 85%/81% | 83% |
| **de Araujo 2019** | 33 | GSE131174 | 100% | 100% | 81% | 93% | NA | 98% |
| **Chen 2017** | 34 | GSE101805 | 79% | 100% | 75% | 100% | 85% | 100% |
| **Huang 2018** | 35 | GSE117563 | 95% | 100% | 80% | 100% | 93% | 100% |
| **Qian 2018** | 36 | GSE103188 | NA | 100% | NA | 100% | 95% | 100% |
| **Huang 2018** | 37 | GSE106953 | 73% | 100% | 96% | 67% | 92% | 100% |
| **Huang 2018** | 38 | GSE106953 | 92% | 67% | 80% | 67% | 93% | 56% |

**Supplementary Table 1 The performance of original models and rebuilt models.**

* The detailed information of references was provided in Supplementary Text 2.

† The performance of model in Pan 2019 referred to diagnose patients with tuberculosis from healthy controls and disease controls, respectively.

‡ The performance of model in Hu 2019 referred to diagnose patients with pulmonary tuberculosis from healthy controls and tuberculous meningitis from healthy controls, respectively.

a: non-available

**Supplementary Text 1 The process of remodeling**

In order to reproduce the original model to the greatest extent, we used the same methods, parameters and training set to rebuild each model. For these parameters which were not provided in original paper, cross validation, grid search or other methods were applied to select optimal parameters. We hold the opinion that these minor alterations were not enough to lead to great differences between original model and rebuilt model, otherwise it indicated that the repeatability of the model was limited, which might influence the subsequent clinical prospective validation in multi-populations and further promotion.

**1. Support vector machine (SVM)**

A total of 4 models were built by SVM. The modeling was realized by “e1071” package in R and the detailed steps were shown as follows:

*>Support vector machine <- svm(group ~ ncRNAs in panel, data = train set, type= "nu-classification", kernel = "linear", cost = 100, cachesize = 5000, tolerance = 0.01, shrinking = FALSE, cross = 3)*

Model optimization (if applicable):

*>Tune <- tune.svm(group ~ ncRNAs in panel, data = train set, gamma = 10^(-6:-1),cost = 10^(1:2))*

*>Adjusted support vector machine <- svm(group ~ ncRNAs in panel, data = train set, gamma=tuned$best.parameters$gamma, cost=tuned$best.parameters$cost)*

Predictive function:

*>Prescore<- predict(Support vector machine, newdata =test set, type = "prob")*

**2. Relevance vector machine (RVM)**

Miotto et al. used 2 methods to build models based on one same panel and one of the modeling methods was RVM. We failed to find available package in R to assist us to rebuild this model and thus “sklearn-rvm” package in python 3.7 was used.

The selection of optimal parameter combination:

*> parameter_selection = GridSearchCV(EMRVR(), parameters, cv=3)*

*> parameter_selection.fit(ncRNAs in panel in train set, group in train set)*

Model building:

*> Relevance vector machine=EMRVR(alpha_max=parameter_selection$best.parameters$alpha_*

*max, gamma= parameter_selection$best.parameters$gamma, kernel="rbf")*

*>Relevance vector machine.fit(ncRNAs in panel in train set, group in train set)*

Predictive function:

*> Relevance vector machine.predict(ncRNAs in panel in test set))*

**3. Logistic regression**

Three models including Pan 2019, Duffy 2018 and Miotto2013-AIC logistic regression were developed by the combination of other algorithms and logistic regression, and 9 models were only constructed by logistic regression according to original papers.

*3.1. Binomial logistic regression*

Zhou 2016, Barry 2018, Qi 2012, Zhang 2013, Alipoor 2019, Chen 2017 and all circRNA diagnostic models except Qian 2018 were rebuilt by “glm” function in R. The processes of modeling were shown as follows:

*>Logistic regression <- glm(group ~ ncRNAs in panel, family = binomial(link = logit), data = train set)*

Predictive function:

*>Pre <- predict(Logistic regression, newdata = test set, type = "response")*

*3.2. Logistic regression with forward stepwise*

Pan 2019 was modeling by logistic regression with forward stepwise and thus the input was as follows:

*>Logistic regression with forward stepwise <- glm(group ~ ncRNAs in panel, family = binomial(link = logit), data = train set)*

*>logit.step <- step(Logistic regression with forward stepwise, direction = "forward")*

The demo of predictive function was described as above.

*3.3 Elastic-net logistic regression*

Duffy 2018 was modeling by elastic-net logistic regression and thus the implementation steps were as follows:

The selection of optimal parameter combination:

*>enet.train <- train(group ~ ncRNAs in panel, data = train set, method = "glmnet", trControl = rainControl(method = "LOOCV"), tuneGrid = expand.grid(.alpha = seq(0,1, by=.2), .lambda = seq(0.00, 0.2, by = 0.02)))*

Model building:

*>enet <- glmnet(ncRNAs in panel, group, family = "binomial", alpha = enet.train$best. parameters*

*$alpha, lambda = enet.train$ best.parameters$ lambda)*

The demo of predictive function was described as above.

*3.4 AIC logistic regression*

Miotto2013-AIC logistic regression was modeling by akaike information criterion (AIC) logistic regression and thus the processes of modeling were as follows:

*>AIC logistic regression <- glm(group ~ ncRNAs in panel, group,family = binomial(link = logit), data = train set)*

*>AIC(AIC logistic regression)*

*>logit.aic=step(AIC logistic regression, trace=0)*

The demo of predictive function was described as above.

**4. Linear combination**

Cui 2017 and Qian 2018 were built by 2 different methods of linear combination, respectively.

*4.1. Linear combination of Cui 2017*

The risk points of Cui 2017 were calculated by the following formula:

$$\mathrm{rsfi}=\sum_{j=1}^{n} Wj * S\mathrm{ij}$$

N is the number of ncRNAs in Cui 2017. S_ij_ represented the ncRNA_j_ on sample i. If the expression level of ncRNA_j_ in tuberculosis patients was less than the lower 5% reference interval of that in healthy controls, S_ij_ was set to 1. Otherwise, S_ij_ was set to 0. W_j_ represented the coefficient of ncRNA_j_ in univariate logistic regression.

The cut-off value was calculated by Yuden index and the prediction results of test sets were obtained by comparing cut-off value and rsf_i_ of each test set.

*4.2. Linear combination of Qian 2018*

The risk points of Qian 2018 were calculated by the following formula:

$$I=\sum_{i=1}^{n} (Ei-Ui)/Ti$$

N is the number of ncRNAs in Qian 2018. E_i_ was the expression level of ncRNA_i_, while the U_i_ and T_i_ were the mean and standard deviation of the expression of ncRNA_i_ in all samples, respectively.

The cut-off value was calculated by Yuden index and the prediction results of test sets were obtained by comparing cut-off value and I of each test set.


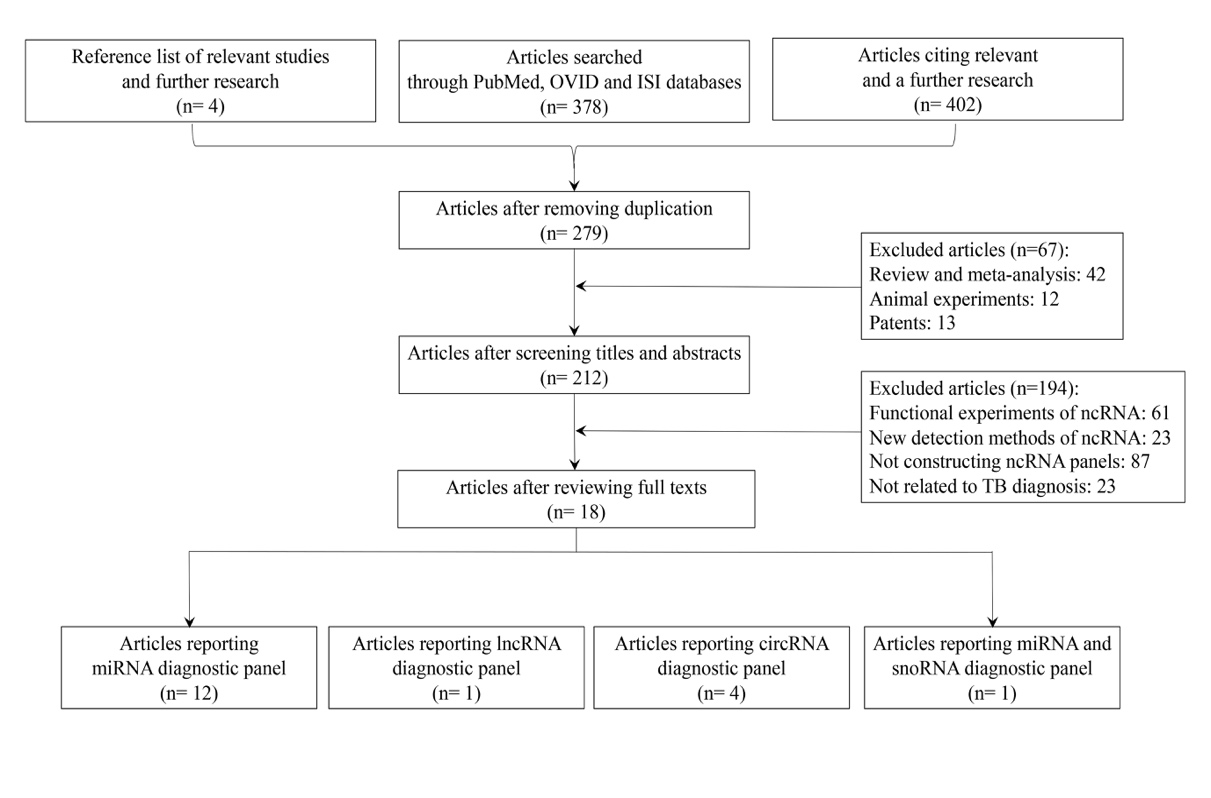


**Supplementary Figure 1 The flow diagram of articles included in this study.** Abbreviations: ncRNA: non-coding RNA; TB: tuberculosis; miRNA: micro RNA; lncRNA: long non-coding RNA; circRNA: circular RNA; snoRNA: small nucleolar RNA.
